# Splicosomal and serine and arginine-rich splicing factors as targets for TGF-β

**DOI:** 10.1186/1755-1536-5-6

**Published:** 2012-04-28

**Authors:** Oskar Hallgren, Johan Malmström, Lars Malmström, Annika Andersson-Sjöland, Marie Wildt, Ellen Tufvesson, Peter Juhasz, György Marko-Varga, Gunilla Westergren-Thorsson

**Affiliations:** 1 Department of Experimental Medical Science, Lund University, Lund, Sweden; 2 Department of Clinical Sciences, Lund, Lund University, Lund, Sweden; 3 Department of Immunotechnology, Lund University, Lund, Sweden; 4 Institute for Molecular Systems Biology, ETH Zurich, Zurich, Switzerland; 5 BG Medicine, 610N Lincoln Street, Waltham, MA, 02451, USA; 6 Department of Electrical Measurement, Lund University, Lund, Sweden

## Abstract

**Background:**

Transforming growth factor-β_1_ (TGF-β_1_) is a potent regulator of cell growth and differentiation. TGF-β_1_ has been shown to be a key player in tissue remodeling processes in a number of disease states by inducing expression of extracellular matrix proteins. In this study a quantitative proteomic analysis was undertaken to investigate if TGF-β_1_ contributes to tissue remodeling by mediating mRNA splicing and production of alternative isoforms of proteins.

**Methodology/Principal findings:**

The expression of proteins involved in mRNA splicing from TGF-β_1_-stimulated lung fibroblasts was compared to non-stimulated cells by employing isotope coded affinity tag (ICAT^TM^) reagent labeling and tandem mass spectrometry. A total of 1733 proteins were identified and quantified with a relative standard deviation of 11% +/− 8 from enriched nuclear fractions. Seventy-six of these proteins were associated with mRNA splicing, including 22 proteins involved in splice site selection. In addition, TGF-β_1_ was observed to alter the relative expression of splicing proteins that may be important for alternative splicing of fibronectin. Specifically, TGF-β_1_ significantly induced expression of SRp20, and reduced the expression of SRp30C, which has been suggested to be a prerequisite for generation of alternatively spliced fibronectin. The induction of SRp20 was further confirmed by western blot and immunofluorescence.

**Conclusions:**

The results show that TGF-β_1_ induces the expression of proteins involved in mRNA splicing and RNA processing in human lung fibroblasts. This may have an impact on the production of alternative isoforms of matrix proteins and can therefore be an important factor in tissue remodeling and disease progression.

## Background

Remodeling of the airway wall, which involves altered extracellular matrix deposition, is an important feature in airway diseases such as asthma and chronic obstructive pulmonary disease (COPD) [1]. This process has been suggested to be associated with aberrant wound healing, dependent on the presence of myofibroblasts [2,3]. Differentiated myofibroblasts can be distinguished from fibroblasts by *de novo* synthesis of α-smooth muscle actin (α-SMA), increased expression of alternatively spliced fibronectin (EDA) and assembly of stress fibers [4]. The growth factor TGF-β_1_ has been shown to play an important role in the differentiation process inducing the expression of alternatively spliced fibronectin, which leads to a subsequent increased expression of α-SMA [5] and other cytoskeletal proteins [6]. In addition, TGF-β_1_ is a potent inducer of various extracellular matrix components such as collagen [7], fibronectin [8], and the proteoglycans: biglycan [9,10] and versican [11-13].

During constitutive and alternative splicing of gene products, splice site selection is regulated by altering initial binding of serine-arginine-rich splicing factors (SR proteins) to pre-mRNA. These factors contain an N-terminal RNA recognition motif that allows binding to pre-mRNA and a C-terminal serine-arginine-rich domain that mediates protein-protein interactions. Different exon-splicing enhancers and silencers are recognized by specific subsets of SR proteins [14], which include SRp20, SRp30a (ASF/SF2) SRp30c, 9 G8, SRp40, SRp55, SRp70, SRp75, and SC35 [15-18]. The ratio of different SR proteins and the presence of exon-splicing enhancers and silencers are factors that influence further assembly of splicosomal proteins. SR proteins generally have nuclear localization but some members such as ASF/SF2, 9 G8, and SRp20 also function as mRNA transporters between nucleus and the cytoplasm [19]. The activity of SR proteins is tightly regulated via dynamic events of phosphorylations and dephosphorylations in different domains of the proteins [20]. The phosphorylation pattern of SR proteins not only influence their activity and function but also play a role in sorting SR proteins within the nucleus [21]. Moreover, hypo-phosphorylation of one domain of SR proteins serves as a nuclear export signal [22].

One important aspect of TGF-β_1_-driven myofibroblast differentiation is the exon inclusion of EDA in fibronectin [23], a process that is not fully understood. However, induced expressions of the splicing factors SRp40, SRp20, or ASF/SF2 have been suggested to stimulate inclusion of EDA suggesting that splice site selection is regulated by quantitative changes in multiple factors [24,25]. Several other matrix molecules have been shown to have different splice variants such as biglycan [26], versican [27], decorin [28], and collagen [29]. The exact role of these splice forms has not yet been established.

To investigate the mechanism of TGF-β_1_-induced alternative splicing, we employed isotope coded affinity tag (ICAT^TM^) reagent labeling and tandem mass spectrometry [30] to identify nuclear proteins and characterize the changes in their expression upon TGF-β_1_ stimulation and focus on proteins involved in the splicing process. We were able to provide a detailed quantitative expression pattern of 76 proteins involved in mRNA splicing and RNA processing. The results showed that TGF-β_1_ altered the relative expression of serine and arginine-rich splicing factors that control splice site selection and promote alternative splicing.

## Results

### TGF-β_1_ induces myofibroblast-like characteristics in fibroblasts

**Figure 1:**
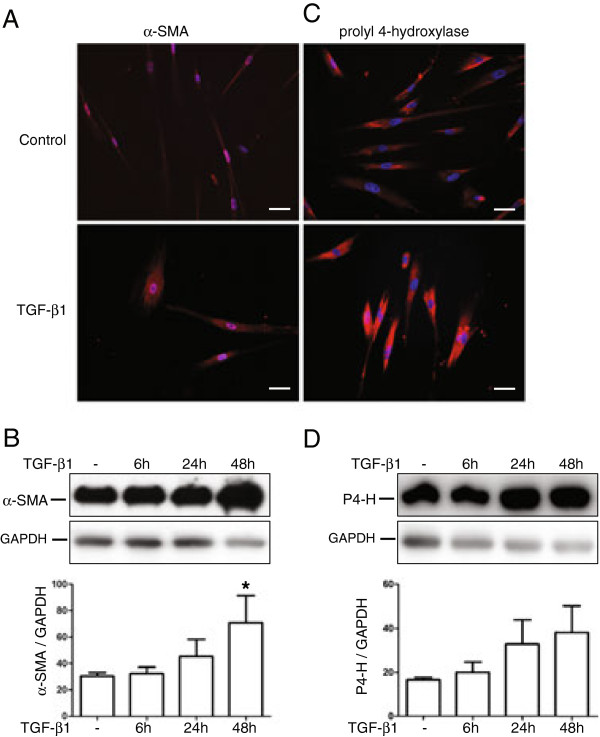
**TGF-β**_**1**_**induces the expression of the α-SMA and prolyl-4-hydroxylase.** Human embryonic lung fibroblasts (HFL-1), incubated with or without TGF-β_1_ (10 ng/mL) for 24 h, were immunostained using antibodies against α-SMA (**A**) and prolyl-4-hydroxylase (**C**). Scale bars represent 50 μm. The α-SMA (**B**) and prolyl-4-hydroxylase (**D**) expression at 6, 24, and 48 h was examined by western blot and bands were quantified with densitometry. Presented values are the intensity of each band relative to loading control: GAPDH. Each value represent mean and SEM from four individual experiments. *P < 0.05.

TGF-β_1_ stimulation for 24 h increased the immuno-reactivity for α-SMA in human embryonic lung fibroblasts (HFL-1) cells, which was accompanied by a change in phenotype as shown by immunofluorescence (Figure 1A). These results were further confirmed by western blot that showed a significant two-fold increase of α-SMA upon TGF-β_1_ stimulation for 48 h (*P* < 0.05) (Figure 1B). Following TGF-β_1_ stimulation HFL-1 cells also had an increased immuno-reactivity for prolyl 4-hydroxylase (Figure 1C), which catalyzes post-translational formation of proline to 4-hydroxyproline in collagens. These results could not fully be confirmed by western blot, which showed a two-fold increase after 48 h (*P* < 0.06) (Figure 1D).

**Figure 2:**
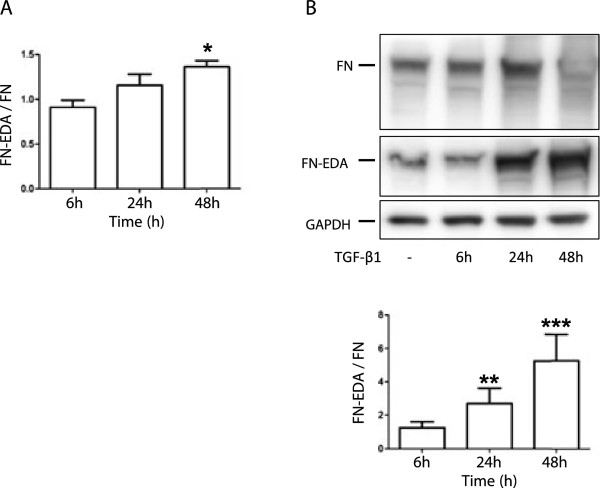
**TGF-β**_**1**_**increases the relative expression of alternative splice forms of fibronectin.** HFL-1 cells were incubated with or without TGF-β_1_ (10 ng/mL) for 6, 24, and 48 h and the relative expression of fibronectin-EDA compared to fibronectin were measured by q-PCR and western blot. (**A**) The relative expression of EDA fibronectin was compared to fibronectin by q-PCR. Each value represents mean and SEM from three individual experiments. (**B**) The relative expression of fibronectin-EDA compared to fibronectin was measured by western blot. Bands were quantified with densitometry and were related the loading control: GAPDH. Each value represents mean and SEM from four individual experiments. *P < 0.05. During the ECL development the 48-h fibronectin band became saturated. *P < 0.05, **P < 0.01, ***P < 0.001.

TGF-β_1_ significantly increased the expression of both fibronectin and EDA fibronectin. However, following TGF-β_1_ stimulation the relative ratio between EDA fibronectin and fibronectin was significantly increased over time as shown by q-PCR (Figure 2A). This was confirmed by western blot showing that TGF-β_1_ increased the relative expression of EDA fibronectin compared to fibronectin from 269% at 24 h to (*P* < 0.01) to 526% at 48 h (*P* < 0.001) (Figure 2B). These data show that TGF-β_1_ may induce myofibroblast differentiation and this is accompanied by an altered splicing pattern, exemplified by the increase in alternatively spliced isoforms of fibronectin.

### Strategy for identification and relative quantification of nuclear proteins

**Figure 3:**
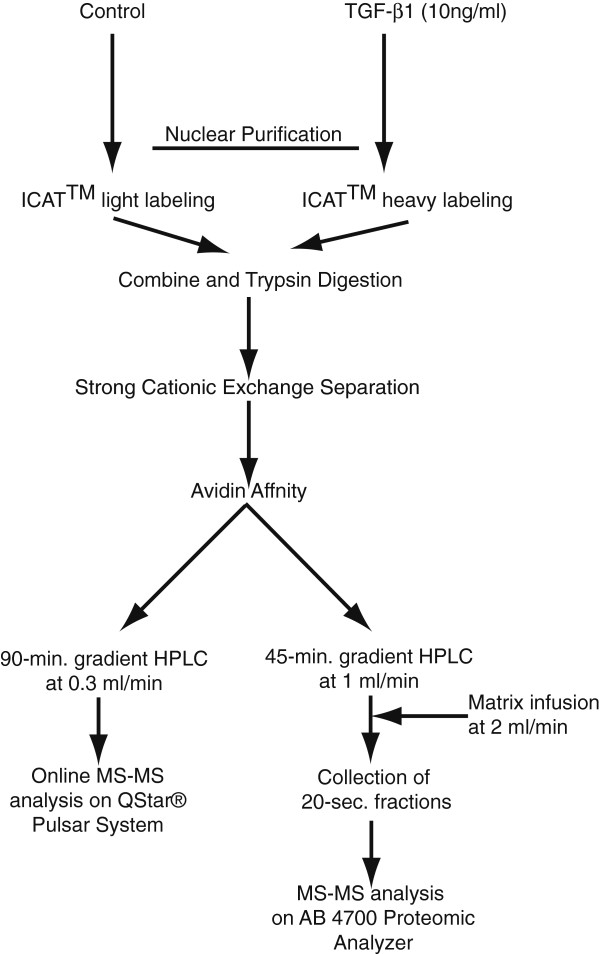
**Schematic illustration of the workflow.** Cell nuclei from TGF-β_1_ stimulated HFL-1 cells were isolated and disrupted by sonication followed by ICAT labeling and trypsin digestion. The tryptic peptides were separated by strong cation exchange chromatography and the ICAT labeled (cysteine-containing) peptides were affinity purified on an avidin column. The captured fractions were split in two. Half of the material was analyzed by online ESI LC-MS/MS on a Qstar^(R)^ Pulsar System and half of the material was analyzed in HPLC coupled to an off-line MALDI target fraction collector and analyzed on AB 4700 Proteomics Analyzer.

**Figure 4:**
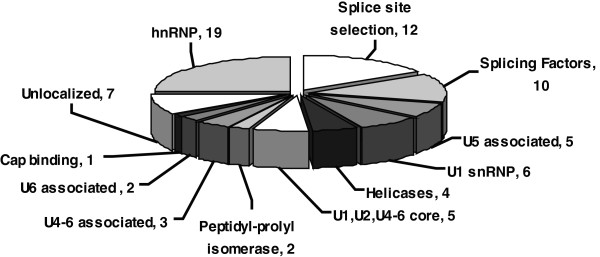
**Classification according to molecular function of the proteins involved in pre-mRNA splicing.** Following TGF-β_1_ stimulation (10 ng/mL) 76 ICAT labeled proteins involved in pre-RNA splicing and RNA processing were identified. The most abundant groups of proteins identified were proteins involved in RNA processing, splicing factors, and other proteins involved in splice site selection.

To address the question whether TGF-β_1_ stimulation affects nuclear proteins that are involved in the splicing process, we employed isotope coded affinity tag (ICAT^TM^) reagent labeling and tandem mass spectrometry. Our experiments showed that the expression of EDA fibronectin increased from 6 to 48 h of TGF-β_1_ stimulation (Figure 2A), which indicates that an alternative splice site selection was induced after 24 h. We therefore decided to examine the expression of nuclear proteins at this time point. Figure 3 shows a schematic overview of the workflow used for the identification and relative quantification of proteins in the nuclear fractions [30-32]. Around 2500 proteins were identified in multiple LC-MS/MS experiments. This list was reduced to approximately 2000 unique proteins after consolidating the peptide-protein assignments. However, approximately 1,700 of these could be quantified after the proper normalization of the relative abundances of ‘heavy’ and ‘light’ ICAT^TM^ reagent labeled peptides. After classifying the proteins based on gene ontology [33], 76 proteins involved in pre-mRNA splicing and RNA processing were selected and divided into 13 functional groups as shown in Figure 4 and in Additional file 1: Table S1. The most abundant group was classified as heterogeneous ribonucleoproteins and contained 19 proteins (hnRNP). The second most abundant group (12 proteins) was proposed to be involved in splice site selection and the third most abundant group, containing 10 proteins, was classified as splicing factors. Nineteen proteins were subunits of the snRNPs or associated with the U1, U5, or U4/U6 snRNPs. In addition, helicases and peptidyl-prolyl cis-isomerases were detected. The relative standard deviation (RSD) was calculated from all ratios of the total list of identified proteins matched by more than one peptide and the average of this error was found to be 11% +/− 8. The proteome wide error was less than 20%, and in the case of the splicing factors the majority of RSDs was less than 20%.

Additional file 1: Table S1 show the relative expression levels (defined as TGF-β_1_ stimulated/control) of the individual splicing related proteins calculated from four unique experiments from independent cell cultures and ICAT workflow runs.

**Figure 5:**
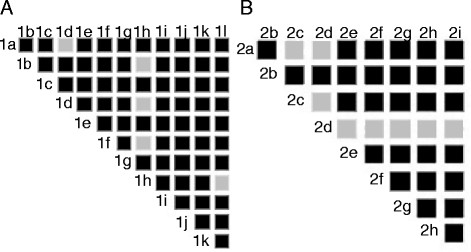
**Relative change of proteins involved in splice site selection.** The relative change of proteins involved in splice site selection and splicing factors following TGF-β_1_ stimulation (10 ng/mL) was calculated. The result, when comparing the relative expression between every protein, is visualized as a color grid. Black box represents no statistically significant difference and light grey represents a significant difference of at least *P* <0.05. 1a denotes: RNA-binding region containing protein 2, 1b: splicing factor, proline-and-glutamine-rich (PTB-associated splicing factor, 1c: splicing factor 3 subunit 1, 1d: splicing factor 3A subunit 3, 1e: splicing factor 3B subunit 1, 1f: U2 small nuclear ribonucleoprotein auxiliary factor 35 Kda subunit related-protein 2, 1 g: splicing factor 3A subunit 2, 1 h: polyadenylate-binding protein 4, 1i: 54 Kda nuclear RNA- and DNA-binding protein (P54(Nrb)), 1j: FUSE binding protein 2, 1 k: splicing factor 3B subunit 3 (spliceosome associated protein 130), and 1 l: splicing factor 3B subunit 793. 2a denotes: splicing factor, arginine/serine-rich 1 (ASF-1), 2b: splicing factor, arginine/serine-rich 7 (splicing factor 9 G8, 2c: splicing factor, arginine/serine-rich 3 (pre-Mrna splicing factor SRP20), 2d: splicing factor U2AF 35 splicing factor U2AF 35 Kda subunit (U2 auxiliary factor 35 Kda subunit), 2e: splicing factor U2AF 65 Kda subunit, 2f: splicing factor, arginine/serine-rich 9 (pre-Mrna splicing factor SRP30c), 2 g: splicing factor, arginine/serine-rich 6 (pre-Mrna splicing factor SRP55), 2 h: splicing factor, arginine/serine-rich 4 (pre-Mrna splicing factor SRP75), 2i: splicing factor arginine/serine-ich 5 (HRS) (pre-Mrna splicing factor SRP40).

Interestingly, TGF-β_1_ altered the expression of splicing factors and additional proteins involved in splice site selection. The expression of splicing factor U2 small nuclear ribonucleoproteins auxiliary factor (65 kDa) (U2AF), and SRp20 were significantly increased by TGF-β_1_ (1.09-fold and 1.24-fold, respectively) (Additional file 1: Table S1). Furthermore, splicing factor SRp30c was significantly repressed (0.79-fold) following TGF-β_1_ stimulation. The relative expression of all proteins involved in splice site selection (grouped as splice site selection and splicing factors in Additional file 1: Table S1) was further statistically analyzed as shown in Figure 5. The data show that TGF-β_1_ significantly repressed the expression of SRp30C labeled ‘2d’ compared to all other splicing factors except SRp 9 G8 (2b). SRp20 (2c) was significantly increased compared to SRp1 (2a) and SRp30c (2d). When comparing the levels of SRp20 with SRp30c, the actual difference between these two splicing factors was 1.6-fold. Several other proteins were significantly changed upon TGF-β_1_ stimulation, such as RNA-binding region containing proteins, U5 snRNP 100 kDa protein, U5 small nuclear ribonucleoprotein 200 kDa, CGI-59 protein, and Cisplatin resistance-associated over expressed protein. In addition the expression of the helicase Nucleolar RNA helicase II (Nucleolar RNA helicase Gu) was also significantly different.

### Western blot analysis of individual SR protein expression

**Figure 6:**
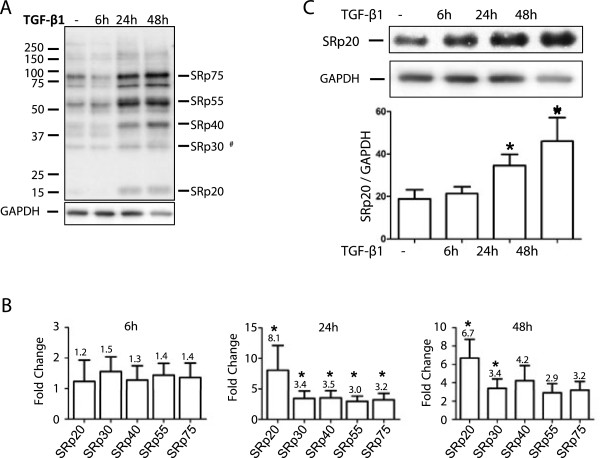
**Western blot analysis of SR proteins after stimulation by TGF-β**_**1**_**.** (**A**) HFL-1 cells were stimulated with TGF-β_1_ (10 ng/mL) for 6, 24, and 48 h. Cell homogenates were separated by SDS-PAGE and after blotting the proteins were detected with an antibody that recognizes a phospho-epitope on a wide range of SR proteins. Bands were visualized by a fluorescent secondary antibody and analyzed on Odyssey® FC imaging system. The most prominent bands represent the splicing factors SRp75, SRp55, SRp40, SRp30a-c, and SRp20. ^#^The band migrating around 30-kDa could consists of several splicing factors with the approximate molecular weight of 30 kDa. (**B**) Bands were quantified by densitometry and were then related to their respective GAPDH loading control. Values show the relative expression between TGF-β_1_-stimulated cells compared to untreated. (**C**) The expression of SRp20 at 6, 24, and 48 h was examined by western blot and bands were quantified with densitometry. The antibody is not directed against phospho-epitope of the protein. Presented values are the intensity of each band relative to the intensity of the loading control: GAPDH. Each value represent mean and SEM from four individual experiments. *P < 0.05.

Phosphorylated SR proteins from TGF-β_1_-stimulated HFL-1 cells were analyzed by western blots using an antibody as previously described [34,35]. This antibody detects a conserved phospho-epitope on SR proteins, which does not coincide with absolute protein levels. We detected a complete array of SR proteins, including SRp75, SRp55, SRp40, SRp20, and an additional band that corresponds to proteins that co-migrate around 30 kDa (ASF/SF2, SC35, or SRp30c) [35] (Figure 6A and Table 1). TGF-β_1_ induced an increase of all the detected SR proteins. A moderate increase was observed at 6 h but at 24 and 48 h the increase was more pronounced (Figure 6A and B). Moreover, TGF-β_1_ triggered a 3.2-fold increase of the SRp75 at 24 h (*P* < 0.05) that was preserved at 48 h. SRp55 was 3.0-fold increased at 24 h (*P* < 0.05) and then slightly decreased at 48 h. SRp40 was continuously increased over time from a 3.5-fold elevation at 24 h (*P* < 0.05) to a 4.2-fold increase at 48 h. There was 3.4-fold increase of the band that was detected at 30 kDa at 24 h (*P* < 0.05) and the same increase was retained at 48 h. SRp20 was elevated 8.1-fold compared to untreated cells at 24 h (*P* < 0.05) which declined to 6.7-fold at 48 h (Figure 6B). To verify the TGF-β_1_-induced increase of SRp20 we performed separate western blots using an antibody that recognizes the actual protein and does not discriminate between phosphorylated and non-phosphorylated variants (Figure 6C). With this antibody there was a 1.8-fold increase of SRp20 at 24 h (*P* < 0.05) and a 2.4-fold increase at 48 h (*P* < 0.05), which supports the results from Additional file 1: Table S1.

**Table 1:** **Comparison of TGF-β**_**1**_-**induced fold change from ICAT and western blot**

	**ICAT**	**Western**^**a**^
SRp20	1,24^b^	8.1^b^
ASF/SF2	0,93	3.4^b,c^
SC35	-	3.4^b,c^
SRp30	0,79	3.4^b,c^
SRp40	1,11	3.5^b^
SRp55	1,01	3.0^b^
SRp75	1,00	3.0^b^

^a^Western blot were performed using an antibody that recognizes a phospho-epitope on SR proteins and not absolute protein levels.

^b^*P* < 0.05.

^c^The band at around 30-kDa could consist of several splicing factors, all with the approximate molecular weight of 30 kDa.

### Cellular localization of SR proteins and SRp20 following TGF-β_1_ stimulation

**Figure 7:**
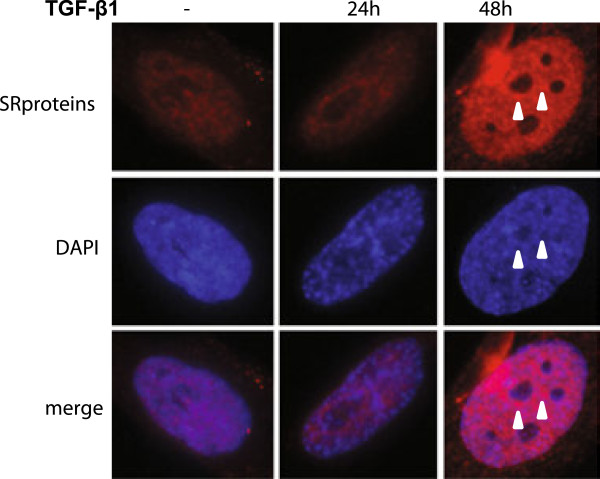
**Cellular localization of SR proteins.** HFL-1 cells were incubated for 24 and 48 h with or without TGF-β_1_ (10 ng/mL). The intracellular localization of SR proteins was examined with immunofluorescence using an antibody that recognizes a phospho-epitope on SR proteins. Staining for SR proteins are shown in red and nucleus are visualized by DAPI (blue). Immune-reactivity for SR proteins was exclusively observed in cell nucleus. Arrowheads show speckles with more intense staining.

Next, we investigated how TGF-β_1_ stimulation influenced the cellular localization of SR proteins and SRp20 by immunofluorescence. The staining pattern of the splicing factors, using the anti-phospho SR proteins antibody, was exclusively nuclear (Figure 7). The staining was more intense at 48 h but this could surprisingly not be observed at 24 h of TGF-β_1_ stimulation compared to untreated cells. In addition the staining at 48 h was more speckled but not to the same extent at 24 h TGF-β_1_ stimulation. The staining when using the SRp20 antibody was mainly nuclear in untreated cells and after 24 h of TGF-β_1_ stimulation although some faint extra-nuclear staining also could be observed ( 8). However, after 48 h TGF-β_1_ stimulation SRp20 was both located intra- and extra-nuclear and in addition the staining was more speckled and intense.

**Figure 8:**
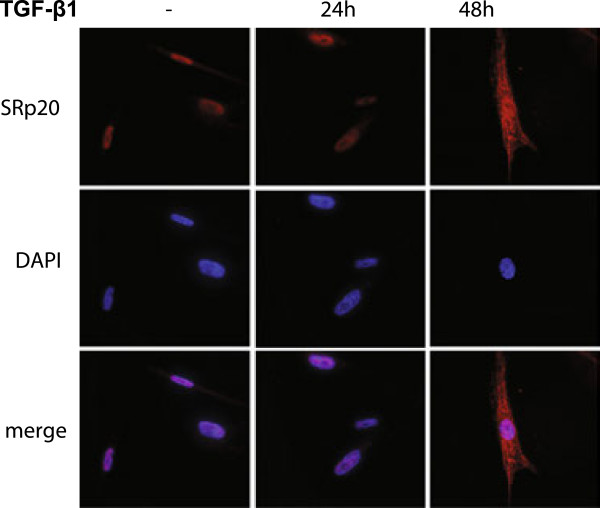
**Cellular localization of SRp20.** HFL-1 cells were incubated for 24 and 48 h with or without TGF-β_1_ (10 ng/mL). The intracellular localization of SRp20 was examined with immunofluorescence using an antibody that is not directed against phospho-epitope of the protein. Staining for SRp20 are shown in red and nucleus are visualized by DAPI (blue).

## Discussion

In the present study the role of TGF-β_1_ on the expression levels of proteins involved in mRNA splicing and RNA processing was examined with a quantitative proteomic approach. We observed that TGF-β_1_ altered the relative ratio of splicing factors that may be important for alternative splicing of proteins. One such example was that TGF-β_1_ affected the levels of splicing factors possibly of importance in EDA fibronectin production, suggesting a regulatory role in myofibroblast differentiation. These results indicate that TGF-β_1_ may contribute to tissue remodeling and disease progression by meditating mRNA splicing and the production of alternative isoforms of proteins.

TGF-β_1_ plays an important role in normal wound healing but is also a central player in the development of fibrosis and tissue remodeling. TGF-β_1_ has been shown to induce the production of various extracellular matrix components, such as collagen [7], fibronectin [8], and proteoglycans [11-13,36], that are elevated during disease. In the present study we verified that TGF-β_1_ triggered a cellular phenotypic switch of lung fibroblasts resulting in a myofibroblast-like phenotype that express α-SMA, as previously described [37]. In addition, TGF-β_1_ induced production of alternative splicing variants of fibronectin. These events were accompanied by alterations in the relative ratios of proteins involved in mRNA splicing and RNA processing. Alternative splicing is an important aspect of gene regulation and results in dramatic biological consequences. It has been estimated that as much as 60% of genes undergo alternative splicing [38] and 15% of human genetic diseases are caused by mutations that destroy functional splice sites or generate new sites [39]. Splicing of mRNA is carried out by the spliceosome, a large nuclear macromolecular complex of five small nuclear ribonucleic particles (snRNPs) and 50–100 polypeptides [40]. In the current study TGF-β_1_ stimulation altered the relative expression of 76 proteins involved in RNA splicing and processing which indicate that TGF-β_1_ may be an important regulator for this process.

The ICAT results were verified by western blots that showed a general increase of phosphorylated SR proteins following TGF-β_1_ stimulation. The increased levels of SRp20, SRp40, and SRp75 from the ICAT experiments were consistent with the western blots (Table 1 and Additional file 1: Table S1). SRp20 was found to be induced by both ICAT and western blot using the antibody detecting the absolute protein level. However, when using the antibody that recognized phospho-epitopes of SRp20 then it was further induced. These data may suggest that TGF-β_1_ may influence the level of phosphorylation of SR proteins which is important for their function and localization. These results were further confirmed with immunofluorescence that indicated an increase of SRp20 accompanied by extra-nuclear localization. Some SR proteins, including SRp20, have in addition to their nuclear functions also been shown to be involved in shuttling mRNA to extra-nuclear localizations [19], which explains the staining pattern. Moreover, when using the antibody that detect phosphorylated SR proteins then the staining was strictly nuclear which may seem contradictory as SRp20 is one of the protein targets for the antibody. However, it has been shown that hypo-phosphorylation is a signal for nuclear export which may explain the difference in staining pattern.

There was a discrepancy in the expression levels of SRp55 as it was induced in the western blots and repressed in the ICAT dataset. A possible explanation for this is that the used antibody is directed against a conserved phospho-epitope and does not coincide with absolute protein levels. In addition, TGF-β_1_ changed the expression of several other proteins such as U5 associated proteins, U5 small nuclear ribonucleoprotein 200 kDa, U5 snRNP 100 kDa protein, and helicases, RNA-dependent helicase p72 and the RNA helicase II/Gu protein, all which have been shown to induce conformational changes in the spliceosome [41-43]. This suggests that TGF-β_1_ also can affect the splicing process via conformational changes of the spliceosome. In addition, we found one member of the hnRNPs family to be repressed, which is of interest since members of the hnRNPs competitively inhibits binding of the splicing factors to the immature mRNA [44]. Splicing of mRNA is a complex operation which involves the activity of multiple proteins. It has been suggested that the relative ratios of proteins involved in selection of splice-sites and splicing factors may be determinants of splice site selection and alternative splicing [45,46]. The experimental setup in this study enabled direct comparison of relative expression levels within these groups of protein (Figure 5). Our data support the idea that relatively small up- and down-regulation of different splicing factors may regulate splicing of a specific mRNA.

The ICAT experiments were chosen at a time-point where the initial changes in fibronectin splicing pattern was observed (t = 24 h) and thus, the quantification of the splicing factors and hnRNPs was made at a time point where alternative spliced sites were selected. We found that the expression of SRp20 was induced and that the expression of SRp30c was repressed. The role of SRp30c in alternative splicing of fibronectin is unclear. However, SRp20 has previously been shown to promote alternative splicing of EDB in fibronectin when induced and to inhibit EDA splicing in chondrocytes [26,47]. In HeLa cells both the levels of EDA + and EDA- fibronectin mRNA were suppressed when over expressing SRp20, [47]. It has been reported in other studies that SRp30a (ASF/SF2) [48,49], 9 G8 [48], and SRp40 [25,47] have positive effects on EDA inclusion. We propose that TGF-β_1_ mediates differential splicing of fibronectin by altering the relative expression and/or phosphorylation pattern of several of these splicing factors, which interact to promote alternative splicing. During the remodeling processes, EDA and EDB are included into the mature mRNA to generate splice isoforms [50]. These alternative splice isoforms promote cell attachment and facilitates cell migration [51] and have been found to be elevated in fibrotic tissue [52] and malignant human tumors [53]. TGF-β_1_ is known to induce an increase of EDA and EDB fibronectin in both fetal and adult fibroblasts [54]. Collectively, these data indicate that TGF-β_1_ not only stimulates myofibroblast differentiation and the expression of ECM proteins, but also may contribute to alternative splicing.

## Conclusions

In summary, we have shown that TGF-β_1_ may alter the expression levels of the splicing factors that regulate alternative splicing of matrix proteins. This may have an impact on the production of alternative isoforms of matrix proteins and suggests a novel mechanism of how TGF-β_1_ mediates tissue remodeling and disease progression.

## Methods

### Cell culturing and TGF-β_1_ stimulation

Human embryonic lung fibroblasts (HFL-1) (ATCC, Manassas, VA, USA) were sub-cultured in Eagles minimum essential medium (EMEM) supplemented with 1% glutamine and 10% fetal calf serum (FCS) and PEST. Before experiments cells were grown to confluency and were starved overnight in Dulbecco’s modified Eagle medium (DMEM) with 0.4% FCS. Cells were washed in PBS and were then incubated with or without TGF-β_1_ (10 ng/mL) (R&D Systems Abingdon, UK) for the time-points indicated in the figures. Experiments were performed in passage 17–22.

### Western blot

Cells were grown under standardized conditions with or without TGF-β_1_ (10 ng/mL) for 6, 24, or 48 h and whole cell lysates were prepared using lysis buffer (50 mM Tris–HCl, 500 mM NaCl, 1% NP-40, 10% glycerol, 10 mM MgCl2, pH 7.4) containing the protein inhibitor cocktail complete mini (1 mM PMSF, 1 μg/mL Aprotinin, 1 μg/mL Pepstatin, 1 μg/mL Leopeptin, Roche, Manheim, Germany). Samples were solubilized in Laemmli’s buffer and equal amounts of total protein (10 μg) were loaded and separated by electrophoresis on 4–12% Bis-Tris Gels (Invitrogen, Gibro, Carlsbad, CA, USA). The proteins were blotted to PVDF membranes (Immobilon-P Transfer Membrane, Millipore Corporation, Billerica, MA, USA). Membranes were incubated with antibodies against: a-SMA (Abcam, Cambridge, UK), prolyl 4-hydroxylase (Acris antibodies, Hiddenhausen, Germany), fibronectin (Dako, Glostrup, Denmark), EDA-fibronectin (Abcam, Cambridge, UK), GAPDH (Santa Cruz Biotechnology, Inc. Santa Cruz, CA, USA), Phospho-SR proteins (Zymed Laboratories, San Francisco, CA, USA), and SRp20 (Zymed Laboratories, San Francisco, CA, USA). Bound antibodies were visualized by peroxidase-conjugated secondary antibodies and enhanced luminescence (Amersham^TM^ western blotting Detection Reagents, GE Healthcare, Uppsala, Sweden) with exception for the blot against phospho-SR proteins where Dy-light 800 nm conjugated secondary antibodies (Cell Signaling Technology Inc., Boston, MA, USA) was used. The luminescence/fluorescence signal was detected on Odyssey® FC imaging system (LI-COR Biosciences, Lincoln, NE, USA). Exposure times were standardized so that all samples were treated the same way for each antibody. Individual bands were quantified with densitometry using the Quantity One software version 4.6.1 (BIORAD Laboratories, Hercules, CA, USA). Data are based on four individual sets of experiments.

### Immunofluorescence

Fibroblasts (7000/well) were grown overnight on chamber slides and were then incubated with or without TGF-β_1_ 24 and 48 h. Cells were then fixed in 4% formaldehyde for 15 min and permeabilized with 0.1% Triton X for 30 min. After blocking in 2% BSA-TBS containing 5% goat serum (Vector laboratories, Burlingame, CA, USA) for 30 min, cells were incubated with primary antibodies against: a-SMA (Abcam, Cambridge, UK), prolyl 4-hydroxylase (Acris antibodies, Hiddenhausen, Germany), Phospho-SR proteins (Zymed Laboratories, San Francisco, CA, USA), and SRp20 (Zymed Laboratories, San Francisco, CA, USA) and with secondary antibodies: Alexafluor 555-conjugated goat anti-mouse antibody and Alexafluor 555-conjugated goat anti-rabbit antibody (both from Molecular Probes Invitrogen, Eugene, OR, USA). To stain nuclei, cells were incubated with DAPI (Molecular Probes Invitrogen, Eugene, OR, USA). Glasses were mounted with mounting media (Dako, Glostrup, Denmark) and photographed using a TE2000-E fluorescence microscope (Nikon, Tokyo, Japan) equipped with a DXM1200C camera (Nikon).

### RNA extraction and cDNA synthesis

Cells were grown under standardized conditions with or without TGF-β_1_ (10 ng/mL) for 6, 24, or 48 h. Total RNA was isolated from cells using RNeasy (Qiagen, GmBH, Hilden, Germany) according to the manufacturer´s instructions. The quantity of RNA was measured by spectrophotometry using a NanoDrop ND-100 (Nano Drop Technologies, Delaware, MD, USA). Total RNA (1 μg) was reverse-transcribed using superscript II according to the manufacturer’s manual (Invitrogen, Carlsbad, CA, USA) and stored at −70°C.

### Real-time RT-PCR

Five μL of cDNA (diluted 1:250) was mixed with 15 μL SYBR-green mixture (PE Biosystems, Foster City, CA, USA) and amplified by real-time RT-PCR using Stratagene MX3005P QPCR system (Agilent Technologies, Santa Clara, CA, USA). Initially the samples were held for 2 min at 50°C then for 10 min at 95°C; they were then cycled for 40 cycles of 30 s at 95°C, 1 min at 58°C, and 1 min at 72°C. Each sample was analyzed in triplicate. Reactions were performed using MX3000P 96-well plates (Agilent Technologies, Santa Clara, CA, USA). All primers were constructed using the online Primer 3 program (http://frodo.wi.mit.edu/primer3/) and they were ordered from A/S DNA Technology (Risskov, Denmark). The following primers were used. Fibronectin forward: CGA TCA CTG GCT TCC AAG TT, and reverse: TCC GAG CAT TGT CAT TCA AG. Fibronectin-EDA forward: AAT CCA AGC GGA GAG AGT CA, and reverse: CGT AAA GGG CTC AGC TCA AG. S18 forward: CGA ACG TCT GCC CTA TCA AC, and reverse: TGC CTT CCT TGG ATG TGG TA. All primers were tested for specificity by sequence alignment in the PubMed nucleic acid database (http://blast.ncbi.nlm.nih.gov/Blast.cgi). Data are based on three individual sets of experiments.

### Purification of nuclei

The nuclei were purified according from a protocol previously described [55]. Briefly, the cells were harvested in 60 mM KCl, 15 mM NaCl, 0.15 mM Spermine, 0.5 mM Spermidine, 15 mM Hepes, and 14 mM Mercaptoethanol supplemented with 0.2% (v/v) Nonidet p40, aprotinin (1 μg/mL), leupeptin (1 μg/mL), pepstatin A (1 μg/mL), PMSF (10 μg/mL) and 0.3 M sucrose. The cell suspension was then homogenized with 60 strokes at 2,000 rpm (Labortechnik, Berlin, Germany) and the homogenate was filtered with 100 μm filter paper (Schleicher&Schuell, Dassel, Germany). The nuclei were counted in a Bürker chamber and the purity was assessed and protein content was determined by Bradford protein reagent kit (Pierce, Rockford, IL, USA).

### Labeling with the acid-cleavable ICAT^TM^ reagent and isolation of cysteine containing peptides

Fibroblast nuclei were lysed in a buffer containing 6 M Guanidium-HCl (pH 8.5), 1% Triton X-100, and 50 mM Tris HCl, followed by sonication. Starting with 500 μg for control and TGF-β_1_ stimulated, each sample was reduced by adding 10 μl of 50 mM TCEP (Tris(2-carboxyethyl)phosphine) and boiled for 10 min. The samples were allowed to cool and the acid cleavable ICAT^TM^ reagent (all the materials in this section are from the ICATTM reagent kit Applied Biosystems, Framingham, MA, USA) was added: the light reagent to the control and heavy reagent containing nine ^13^ C to the TGF-β_1_-treated sample. Alkylation was allowed to complete for 2 h, at 37°C. The two samples are combined and acetone precipitated in order to remove the guanidium-HCl and the unreacted ICAT^TM^ reagent. The pellet was dissolved in a buffer consisting of 50 mM Tris (pH 8.5), 5 mM CaCl_2_, and 10% acetonitrile. Trypsin was added at a 1:40 enzyme/substrate ratio in two additions once at the start of the digestion and once more, 2 h later. Digestion was completed at 37°C overnight.

Following digestion the sample was diluted into 25% acetonitrile, pH 3.0 in order to reduce the buffer concentration below 10 mM. The resulting peptide mixture was injected to a PolyLC (4.6 × 100 mm Polysulfoethyl A) SCX column on Vision workstation (Applied Biosystems, Framingham, MA, USA) at a flow rate of 1 mL/min using a binding buffer (buffer A) 10 mM KH_2_PO_4_, 25% acetonitrile, pH 3 and an elution buffer (buffer B) of 350 mM KCL, 10 mM KH_2_PO_4_, 25% acetonitrile, pH 3. The gradient was 0% B to 10% B in 2 min, 20% B in 15 min, 45% B in 3 min, 100% B in 10 min, and an additional 8 min hold at 100% B. Twenty-three SCX fractions of 1.5 mL volume were collected.

The pH was adjusted for each SCX fraction to 7.2 with 10× PBS buffer (pH 10) and injected to the avidin affinity column as prescribed by the manufacturer (Applied Biosystems, Framingham, MA, USA). Cysteine-containing peptides bound to the avidin column were washed three times: 1 mL of Wash 1, 1 mL of Wash 2, and 1 mL of de-ionized water. Elution of the cysteine-containing peptides was performed with 800 μL of avidin elution buffer. The samples were then concentrated by a speed-vac to dry, cleaved with the *ICAT*^*tm*^ cleaving reagent containing 95% TFA and concentrated again to remove the cleaving reagent. The samples were then taken up in 2% acetonitrile, 0.1% TFA for re-injection for a nanoHPLC MS and MS/MS analysis.

### Protein identification and quantification by LC-MS and -MS/MS

Avidin purified SCX fractions were subjected to MALDI and ESI HPLC-MS and MS/MS analysis in a 50:50 split. For the MALDI based workflow, these fractions were injected in the HPLC loading buffer onto a 100 μm × 15 mm C_18_ Magic column (Auburn, Michrom Bioresources, CA, USA) using a CapTrap pre-column (Auburn, Michrom Bioresources, CA, USA). Mobile phase A was 2% acetonitrile, 0.1% TFA, mobile phase B was 85% acetonitrile, 5% n-PrOH, 10% water, 0.1% TFA. HPLC elution was carried out at a 1-μL/min flow rate on an Ultimate nanoHPLC workstation (Dionex-LC Packings, Hercules, CA, USA). The HPLC elution from the column was collected at 20-s intervals on the MALDI plate using a Probot fraction collector (Dionex-LC Packings). Forty-eight-min HPLC elution was collected to the MALDI plates as an array of 12 × 12 sample spots. The HPLC eluent was mixed with the MALDI matrix (7.5 mg/mL α-cyano-4-hydroxycinnamic acid dissolved in 60:40 acetonitrile-water containing 0.15 mg/mL dibasic ammonium-citrate) through a mixing tee (Upchurch, WA, USA) at a flow rate of 2-μL/min. The most abundant, middle-fractions of the SCX separation were spotted onto two plates using a 90-min HPLC gradient.

MALDI plates were analyzed in automated mode on the AB4700 Proteomics Analyzer (Applied Biosystems, Framingham, MA, USA). First the MS spectra were collected from the entire HPLC run. Then, using an in-house developed program, MS/MS precursors were selected by applying an exclusion algorithm to eliminate: (a) redundant precursors carrying over multiple HPLC fractions; and (b) using only the more abundant members of peptide pairs for MS/MS analysis. MS/MS spectra were acquired using up to 2,500 laser shots/precursor unless the predefined signal-to-noise level in the MS/MS acquisitions was achieved sooner. The MS/MS data were submitted for database searching as a batch to Mascot (http://www.matrixscience.com) through its automation interface of Mascot (Mascot Daemon). The non-redundant NCBI protein database was used. A detailed description of the acceptance criteria for the database searching results will be described elsewhere. In brief, tryptic peptides containing arginine residues were accepted at a Mascot score >20, peptides not containing arginine at a Mascot score >25. Peptides with less-than-significant Mascot score were thoroughly inspected, considering the correlation of peptide basicity with SCX fraction numbers, presence of characteristic high-energy CID fragments, and accurate mass measurements through internal calibration of the MS spectra using the masses of confidently identified peptides as internal mass references.

ESI based LC-MS/MS analyses were carried out using an Ultimate nanoHPLC system (Dionex-LC Packings, CA, USA) on a 75 μM × 150 mm Picofrit C18 column at a 300-nl/min flow rate with a gradient of 5% B to 30% B over 60 min. Mobile phase A was 2% acetonitrile, 0.1% formic acid, mobile phase was 85% acetonitrile, 5% n-PrOH, 10% water, 0.1% formic. A CapTrap (Michrom Bioresources, CA, USA) precolumn was used to preconcentrate the sample.

A platinum electrode was placed behind the HPLC column using a microtee (Upchurch, WA, USA) in order to apply the electrospray voltage (3 kV) for LC-MS/MS analysis. LC-MS/MS was performed using a QSTAR quadrupole time-of-flight instrument (Applied Biosystems, CA, USA). The instrument was set up to perform a 1-s MS scan (300–1500 Da) followed by three most abundant components (two to four charges were allowed) exceeding an intensity threshold of 35 counts. To improve the protein coverage the samples were halved and the analysis was replicated. LC-MS and MS/MS analyses were processed by the ProICAT software (Applied Biosystems, Foster City, CA, USA). Database search results were accepted at >95% confidence interval. Database searching and quantification results from multiple MS platforms were consolidated by parsing all the qualitative and quantitative peptide results into an Oracle database. The set of all the identified peptides were assigned to a minimum set of proteins that could explain all the confidently identified peptide sequences. The abundance ratios of peptide pairs labeled with the heavy (9 × ^13^ C) and light (0 × ^13^ C) ICAT^TM^ reagents were normalized to the median of all peptide ratios in order to generate normalized expression ratios. Expression ratios of peptides matching to the same protein were averaged to generate expression ratios at the protein level.

### Statistical methods

Data are expressed as mean ± SEM. Student’s *t*-test was used to evaluate the differences of the means between groups. Differences were considered significant at *P* < 0.05. All analyses were performed using GraphPad Prism software version 4.00 (GraphPad Software, San Diego, CA, USA).

## Competing interests

The authors declare that they have no competing interests.

## Authors’ contributions

OH, JM, AAS, MW, LM, ET, PJ, GMV, and GWT conceived and designed the experiments; OH, JM, LM, AAS, MW, GMV, and GWT performed the experiments; OH, JM, LM, AAS, MW, GMV, and GWT analyzed the data; OH, JM, LM, ET, GMV, PJ, and GWT contributed materials; OH, JM, AAS, and GWT wrote the paper. All authors read and approved the final manuscript.

## Supplementary Material

Additional file 1 Table S1. Identified and quantified proteins involved in the splicing process Click here for file
