# Supplementary material for: Splicosomal and serine and arginine-rich splicing factors as targets for TGF-β
Source: Fibrogenesis Tissue Repair. 2012 Apr 28;5:6. doi: 10.1186/1755-1536-5-6 (PMC3472233; doi:10.1186/1755-1536-5-6)
Supplement: Additional file 1 — Table S1. Identified and quantified proteins involved in the splicing process [file 1755-1536-5-6-S1.docx]

**Supplemental Table 1: Identified and quantified proteins involved in the splicing process**

| **Protein Name** | **Acc #^a^** | **Ratio^c^** | **STDEV** | **p-value** | **#-pep.** | **Unique Exp.** | **In Fig. 5** |
| --- | --- | --- | --- | --- | --- | --- | --- |
| ***Splice site selection*** |  |  |  |  |  |  |  |
| **RNA-binding region containing protein 2** | **Q14498^b^** | **1,17** | **0,18** | **0,05** | **8** | **4** | **1a** |
| Splicing factor, proline-and glutamine-rich (PTB-associated splicing factor) | P23246^b^ | 0,98 | 0,03 | 0,48 | 2 | 1 | 1b |
| Splicing factor 3 subunit 1 | Q15459^b^ | 1,02 | 0,20 | 0,90 | 2 | 2 | 1c |
| Splicing factor 3A subunit 3 | Q9D554^b^ | 0,96 | 0,17 | 0,58 | 5 | 3 | 1d |
| Splicing factor 3B subunit 1 | O57683^b^ | 1,06 | 0,21 | 0,42 | 8 | 4 | 1e |
| U2 small nuclear ribonucleoprotein auxiliary factor 35 kDa subunit related-protein 2 | AAH50451.1 | 0,90 |  |  | 1 | 1 | 1f |
| Splicing factor 3A subunit 2 | Q15428 | 1,01 | 0,38 | 0,99 | 2 | 2 | 1g |
| Polyadenylate-binding protein 4 | Q13310 | 1,20 | 0,04 | 0,08 | 2 | 1 | 1h |
| 54 kDa nuclear RNA- and DNA-binding protein (p54(nrb)) | Q15233^b^ | 1,16 | 0,11 | 0,14 | 3 | 3 | 1i |
| FUSE binding protein 2 | AAC50892.1 | 1,18 | 0,28 | 0,29 | 4 | 2 | 1j |
| Splicing factor 3B subunit 3 (Spliceosome associated protein 130) | Q15393^b^ | 1,13 | 0,24 | 0,25 | 6 | 3 | 1k |
| Splicing factor 3B subunit 793 | Q9BWJ5 | 1,03 |  |  | 1 | 1 | 1l |
|  |  |  |  |  |  |  |  |
| ***Splicing Factors*** |  |  |  |  |  |  |  |
| Splicing factor, arginine/serine-rich 1 (ASF-1) | Q07955 | 0,99 | 0,19 | 0,89 | 11 | 4 | 2a |
| Splicing factor, arginine/serine-rich 7 (Splicing factor 9G8) | Q16629^b^ | 0,99 | 0,22 | 0,94 | 5 | 3 | 2b |
| **Splicing factor, arginine/serine-rich 3 (Pre-mRNA splicing factor SRP20)** | **P23152** | **1,24** | **0,23** | **0,03** | **7** | **4** | 2c |
| Splicing factor U2AF 35Splicing factor U2AF 35 kDa subunit (U2 auxiliary factor 35 kDa subunit) | Q01081 | 1,27 | 0,22 | 0,17 | 3 | 2 | 2d |
| **Splicing factor U2AF 65 kDa subunit** | **P26368^b^** | **1,09** | **0,10** | **0,01** | **14** | **4** | 2e |
| **Splicing factor, arginine/serine-rich 9 (Pre-mRNA splicing factor SRp30C)** | **Q13242** | **0,79** | **0,06** | **0,01** | **6** | **2** | 2f |
| Splicing factor, arginine/serine-rich 6 (Pre-mRNA splicing factor SRP55) | AAA93072.1 | 1,01 |  |  | 1 | 1 | 2g |
| Splicing factor, arginine/serine-rich 4 (Pre-mRNA splicing factor SRP75) | AAA36649.1 | 1,00 | 0,10 | 0,95 | 3 | 2 | 2h |
| Splicing factor arginine/serine rich 5 (HRS) (Pre-mRNA splicing factor SRP40) | AAC39543.1 | 1,11 |  |  | 1 | 1 | 2i |
| 38kDa splicing factor; SPF 38 [Homo sapiens] | gi\|3746837 | 1,14 | 0,19 | 0,49 | 2 | 1 |  |
|  |  |  |  |  |  |  |  |
| ***U5 associated*** |  |  |  |  |  |  |  |
| U5 snRNP-associated 102 kDa | O94906^b^ | 1,08 | 0,27 | 0,60 | 4 | 2 |  |
| **U5 snRNP 100 kD protein** | **AAH02366.1** | **1,17** | **0,17** | **0,02** | **8** | **3** |  |
| **U5 small nuclear ribonucleoprotein 200 kDa helicase** | **gi\|14724649** | **1,23** | **0,40** | **0,03** | **17** | **4** |  |
| 116 kDa U5 small nuclear ribonucleoprotein component | Q15029^b^ | 1,24 | 0,72 | 0,17 | 18 | 4 |  |
| U5 snRNP-specific protein; U5 snRNP-specific protein (220 kD) | gi\|2463576^b^ | 1,38 | 0,54 | 0,14 | 6 | 3 |  |
|  |  |  |  |  |  |  |  |
| ***U1 snRNP*** |  |  |  |  |  |  |  |
| U1 small nuclear ribonucleoprotein C | P09234 | 0,82 |  |  | 1 | 1 |  |
| Fas-ligand associated factor 1 | AAB93495.1 | 0,87 |  |  | 1 | 1 |  |
| U1 small nuclear ribonucleoprotein 70 kDa (U1 snRNP 70 kDa) (snRNP70) (U1-70K) | P08621 | 1,07 | 0,16 | 0,46 | 4 | 3 |  |
| U1 small ribonucleoprotein 1SNRP homolog | AAC97961.1^b^ | 1,15 | 0,19 | 0,30 | 3 | 2 |  |
| **CGI-59 protein** | **AAD34054.1** | **1,20** | **0,13** | **0,00** | **8** | **3** |  |
| **Cisplatin resistance-associated overexpressed protein** | **AAH47043.1** | **1,28** | **0,09** | **0,04** | **3** | **3** |  |
|  |  |  |  |  |  |  |  |
| ***Helicases*** |  |  |  |  |  |  |  |
| Probable ATP-dependent RNA helicase p47 | Q13838^b^ | 1,09 | 0,29 | 0,29 | 14 | 4 |  |
| RNA helicase Nr.2 | AAD43033.1 | 1,11 | 0,36 | 0,58 | 4 | 3 |  |
| ATP-dependent helicase DHX8 (RNA helicase HRH1) | AAH47327.1 | 1,36 |  |  | 1 | 1 |  |
| **Nucleolar RNA helicase II (Nucleolar RNA helicase Gu)** | **AAF78930.2** | **1,57** | **0,50** | **0,02** | **7** | **3** |  |
|  |  |  |  |  |  |  |  |
|  |  |  |  |  |  |  |  |
| **Protein Name** | **Acc #^a^** | **Ratio^c^** | **STDEV** | **p-value** | **#-pep.** | **Unique Exp.** | **In Fig. 5** |
| ***U1,U2,U4-6 core*** |  |  |  |  |  |  |  |
| Splicing coactivator subunit SRm300 RNA binding protein | AAF21439.1 | 0,74 | 0,28 | 0,10 | 5 | 2 |  |
| Small nuclear ribonucleoprotein F (snRNP-F) | Q15356 | 1,06 | 0,09 | 0,37 | 3 | 2 |  |
| Small nuclear ribonucleoprotein Sm D3 | P43331^b^ | 1,09 | 0,21 | 0,34 | 8 | 4 |  |
| **Small nuclear ribonucleoprotein Sm D2** | **P43330^b^** | **1,15** | **0,06** | **0,00** | **6** | **2** |  |
| Similar to Small nuclear ribonucleoprotein Sm D2 | XP_061427.1 | 1,25 |  |  | 1 | 1 |  |
|  |  |  |  |  |  |  |  |
| ***Peptidyl-prolyl isomerase*** |  |  |  |  |  |  |  |
| Peptidyl-prolyl cis-trans isomerase H (PPIase H) | O43447 | 0,97 |  |  | 1 | 1 |  |
| Peptidyl-prolyl cis-trans isomerase G PPIG protein | Q13427 | 1,09 | 0,35 | 0,41 | 11 | 4 |  |
|  |  |  |  |  |  |  |  |
| ***U4-6 associated*** |  |  |  |  |  |  |  |
| U4/U6 small nuclear ribonucleoprotein Prp3 | O43395 | 0,79 |  |  | 1 | 1 |  |
| U4/U6 small nuclear ribonucleoprotein Prp4 | AAB87640.1 | 0,94 |  |  | 1 | 1 |  |
| U4/U6.U5 tri-snRNP-associated 65 kDa protein | gi\|13926070 | 0,97 | 0,18 | 0,85 | 2 | 2 |  |
|  |  |  |  |  |  |  |  |
| ***U6 associated*** |  |  |  |  |  |  |  |
| U6 snRNA-associated Sm-like protein LSm4 (Glycine-rich protein) (GRP) | Q9Y4Z0 | 1,00 |  |  | 1 | 1 |  |
| U6 snRNA-associated Sm-like protein LSm7 | AAC25622.1 | 1,05 |  |  | 1 | 1 |  |
|  |  |  |  |  |  |  |  |
| ***Cap binding*** |  |  |  |  |  |  |  |
| 80 kDa nuclear cap binding protein (NCBP 80 kDa subunit) | Q09161 | 1,41 |  |  | 1 | 1 |  |
|  |  |  |  |  |  |  |  |
| ***Unlocalized*** |  |  |  |  |  |  |  |
| Suppressor of SWI4 1 homolog (Ssf-1) (Peter Pan homolog) | Q9NQ55 | 0,87 | 0,13 | 0,23 | 3 | 2 |  |
| Ser/Arg-related nuclear matrix protein | gi\|3005586 | 0,88 |  |  | 1 | 1 |  |
| Cell division control protein 5 | gi\|2887435 | 0,94 | 0,08 | 0,46 | 2 | 2 |  |
| Nucleolar protein family A | AAH00009.1 | 1,04 | 0,12 | 0,64 | 3 | 1 |  |
| Splicing factor 1 (Zinc finger protein 162) (Transcription factor ZFM1) | AAB03514.1 | 1,14 | 0,25 | 0,56 | 2 | 2 |  |
| **Serine/threonine-protein kinase PRP4 homolog** | **AAH34969.1** | **1,26** | **0,21** | **0,01** | **8** | **2** |  |
| Splicing factor 45 | AAH09064.1 | 1,51 |  |  | 1 | 1 |  |
|  |  |  |  |  |  |  |  |
| ***hnRNP*** |  |  |  |  |  |  |  |
| RNA-binding protein 5 (RNA binding motif protein 5) | AAA99715.1 | 0,65 |  |  | 1 | 1 |  |
| Autoantigen p542 | AAC28898.1 | 0,81 |  |  | 1 | 1 |  |
| Heterogenous nuclear ribonucleoprotein U (hnRNP U) | Q00839^b^ | 0,94 | 0,20 | 0,15 | 27 | 4 |  |
| **Heterogeneous nuclear ribonucleoprotein D0 (hnRNP D0)** | **Q14103^b^** | **0,96** | **0,12** | **0,08** | **28** | **4** |  |
| Heterogeneous nuclear ribonucleoprotein A/B | Q99729 | 0,96 | 0,13 | 0,30 | 15 | 2 |  |
| Heterogeneous nuclear ribonucleoprotein F | P52597^b^ | 0,97 | 0,02 | 0,23 | 2 | 2 |  |
| Heterogeneous nuclear ribonucleoprotein homolog JKTBP | gi\|14723537 | 0,98 | 0,18 | 0,68 | 16 | 3 |  |
| Heterogeneous nuclear ribonucleoproteins C1/C2 | Q9Z204 | 0,98 | 0,14 | 0,88 | 2 | 1 |  |
| Heterogeneous nuclear ribonucleoproteins A2/B1 | P22626^b^ | 1,01 | 0,15 | 0,88 | 6 | 2 |  |
| Polypyrimidine tract-binding protein 1 (PTB) | CAA46443.1 | 1,03 |  |  | 1 | 1 |  |
| Heterogeneous nuclear ribonucleoprotein L | P14866^b^ | 1,05 | 0,25 | 0,54 | 12 | 4 |  |
| Heterogeneous nuclear ribonucleoprotein Q (hnRNP Q) | O60506 | 1,07 | 0,16 | 0,17 | 10 | 3 |  |
| Heterogeneous nuclear ribonucleoprotein M (hnRNP M) | P52272 | 1,10 | 0,25 | 0,10 | 19 | 4 |  |
| **Heterogeneous nuclear ribonucleoprotein H (hnRNP H)** | **P31943^b^** | **1,10** | **0,18** | **0,02** | **20** | **4** |  |
| **Heterogeneous nuclear ribonucleoprotein R** | **O43390^b^** | **1,10** | **0,14** | **0,00** | **20** | **4** |  |
| Heterogeneous nuclear ribonucleoprotein A1 | P49312^b^ | 1,10 | 0,19 | 0,10 | 11 | 4 |  |
| Heterogeneous nuclear ribonucleoprotein A3 (hnRNP A3) | P51991 | 1,11 | 0,21 | 0,19 | 8 | 4 |  |
| Heterogeneous nuclear ribonucleoprotein K (hnRNP K) | Q07244 | 1,14 | 0,20 | 0,14 | 6 | 4 |  |
| Heterogeneous nuclear ribonucleoprotein A | Q13151 | 1,55 | 0,38 | 0,29 | 2 | 2 |  |

^a^NCBI ([www.ncbi.nlm.nih.gov](http://www.ncbi.nlm.nih.gov)) or Swissprot ([www.expasy.org](http://www.expasy.org)) accession numbers

^b^Present in gel based separation methods published previously

^c^The expression ratio of proteins from four unique experiments were averaged from the intensity ratios of peptide pairs (light ICAT^TM^ reagent labeled peptides from non-stimulated cells and heavy ICAT^TM^ reagent-labeled peptides from TGF-β__ stimulated cells).

**^d^**STDEV denotes standard deviation

^e^#-pep denotes the total number of identified cysteine-containing peptides in each identified protein. In cases where more than one cysteine-containing peptide was matched to the protein, the standard deviation from multiple independent observations was calculated as well as the p-value, to indicate the consistency of the quantification results.

^f^ Unique Exp. denotes the number of experiments where each protein was detected.
